# The Nose

**Published:** 1890-07

**Authors:** 


### THE NOSE.

The mouth is not more distinctly the gateway to the alimentary system than the nose is to the respiratory, nor is it more carefully designed for preparing food to enter the stomach, than is the nose for preparing air to enter the lungs.

It is important for all persons to be instructed that the nose has three highly necessary functions related to breathing, and for which it is delicately adapted, besides those of an opening for air and a detective of bad air, viz. : to warm, to moisten, and to filter, the air which it admits ; and that neither of these functions can be performed by the mouth. However cold or warm the atmospheric temperature, the air is brought almost, if not quite, to the temperature of the blood in passing through the nose alone, and even before reaching the pharynx, or cavity back of the nose ; that however dry the external air may be, it is completely saturated with moisture by passing through the nose ; that instead of the moisture of the breath being supplied by and carried from the pulmonary tissues, it is carried to them from the nose, and may be deposited as a dew upon the bronchial surfaces ; that gaseous exchanges take place in the nose, between the gases of the blood and those of the air, just as in the lungs ; that nearly one-fiftieth of the exhaled carbonic acid is given off by the nasal mucous membrane, even when expiration is not performed through the nose.

It is evident that if air of a low temperature be brought in contact with the lower respiratory passages, inflammatory processes would be likely to be induced ; and when at the same time the air is dry, if not duly moistened by being inhaled through the nose, this tendency would be greatly increased, and the co-incidence must be the most efficient occasion of consumption, pneumonia, bronchitis, etc. Drying out thus the moisture of the air-passages, provokes an exudation of the albuminous part of the blood, and so provides the best possible culture fluid for the bacillus tuberculosis, the pneumonia, typhoid, or other health destroying germs to lodge and propagate.

In the mouth there is no provision for supplying sufficient moisture. Deprived of its normal watery constituents, the normal mucus of the bronchi becomes thick and a source of irritation.

By the abattis of hairs at its entrance, and by its narrow and tortuous mucous passages, the nose protects the parts below not only from the irritating qualities of particles of dust and smoke, but often from the deadly invasions of microbes. With all parts of the current of inspired air coming in immediate contact with the nasal mucous membrane, it must follow that vast numbers of germs will adhere to this membrane.

On the one hand is the narrow entrance, through which the air enters with all its invading hosts. On the other hand, Nature has provided for the defense of this grand point of attack, a tortuous defile, which the current of air cannot pass through without hugging its sides and its warm and moist lining. In this lining are ever watchful defenders, congregated to devour the army of invading germs. Here, then, is continuously waged one of the great battles of life.

From what has been said, the following conclusions can be drawn :

1. The nose should be kept clean.

2. All obstructions to nasal respiration should be removed. Especially should the mouth be kept firmly closed on emerging into the cold air ; and while it should always be used as little as may be convenient, for breathing, let such use be carefully avoided in the presence of foul, smoky, foggy, dusty or extra dry and cold air.

